# Genotype-independent plant transformation

**DOI:** 10.1093/hr/uhac047

**Published:** 2022-03-14

**Authors:** Nathan A Maren, Hui Duan, Kedong Da, G Craig Yencho, Thomas G Ranney, Wusheng Liu

**Affiliations:** Department of Horticultural Science, North Carolina State University, Raleigh, NC, 27607, USA; USDA-ARS, U.S. National Arboretum, Floral and Nursery Plants Research Unit, Beltsville Agricultural Research Center (BARC)-West, Beltsville, MD 20705, USA; Department of Horticultural Science, North Carolina State University, Raleigh, NC, 27607, USA; Department of Horticultural Science, North Carolina State University, Raleigh, NC, 27607, USA; Mountain Crop Improvement Lab, Department of Horticultural Science, Mountain Horticultural Crops Research and Extension Center, North Carolina State University, Mills River, NC 28759, USA; Department of Horticultural Science, North Carolina State University, Raleigh, NC, 27607, USA

## Abstract

Plant transformation and regeneration remain highly species- and genotype-dependent. Conventional hormone-based plant regeneration via somatic embryogenesis or organogenesis is tedious, time-consuming, and requires specialized skills and experience. Over the last 40 years, significant advances have been made to elucidate the molecular mechanisms underlying embryogenesis and organogenesis. These pioneering studies have led to a better understanding of the key steps and factors involved in plant regeneration, resulting in the identification of crucial growth and developmental regulatory genes that can dramatically improve regeneration efficiency, shorten transformation time, and make transformation of recalcitrant genotypes possible. Co-opting these regulatory genes offers great potential to develop innovative genotype-independent genetic transformation methods for various plant species, including specialty crops. Further developing these approaches has the potential to result in plant transformation without the use of hormones, antibiotics, selectable marker genes, or tissue culture. As an enabling technology, the use of these regulatory genes has great potential to enable the application of advanced breeding technologies such as genetic engineering and gene editing for crop improvement in transformation-recalcitrant crops and cultivars. This review will discuss the recent advances in the use of regulatory genes in plant transformation and regeneration, and their potential to facilitate genotype-independent plant transformation and regeneration.

## Introduction

Plant transformation and regeneration are highly species- and genotype-dependent and are often the principal bottlenecks in applying genetic engineering and gene editing for crop trait improvement [1–4]. Plant transformation starts with delivering genes of interest into single regeneration-competent or embryogenic stem cells, typically achieved through *Agrobacterium*-mediated or biolistics-based methods. *In vitro* plant regeneration is a process of generating a whole plant from a single cell derived from various explants such as leaf, cotyledon, hypocotyl, root, microspore, and immature embryo, and usually involves the formation of callus from explants cultured on a callus-inducing medium (CIM). Callus is a highly heterogeneous group of cells with organized structures similar to lateral root primordia [5], most of which are regeneration-incompetent cells with a limited number of cells capable of proliferating. The foundation of plant regeneration lies in the totipotency of plant cells, which is the ability of a somatic or meristematic cell to regenerate into an entire plant [6]. The successful regeneration of a singular transformed cell into a fully functioning plant dictates the success of plant transformation.

Regeneration-competent cells can originate from the proliferation of pre-existing undifferentiated meristematic cells within explants that will go through direct organogenesis to develop into plantlets. This process permits the co-cultivation of explants with *Agrobacterium* for the delivery of the genes of interest into the regeneration-competent cells, e.g. the *Agrobacterium*-mediated transformation of cotyledonary nodal regions of soybean where axillary meristem is located. In many plant species, however, regeneration-competent cells originate from reprogrammed differentiated somatic cells via a dedifferentiation process to regain the capacity for proliferation competence or pluripotency, i.e. the ability of plant embryogenic or stem cells to develop into all shoot and root cell types [7]. Acquisition of pluripotency converts somatic cells to regeneration-competent cells for callus induction. Thus, *Agrobacterium*-mediated gene delivery can be performed at different stages from isolated explants such as leaf discs or immature embryos to the resulting callus.

**Figure 1:**
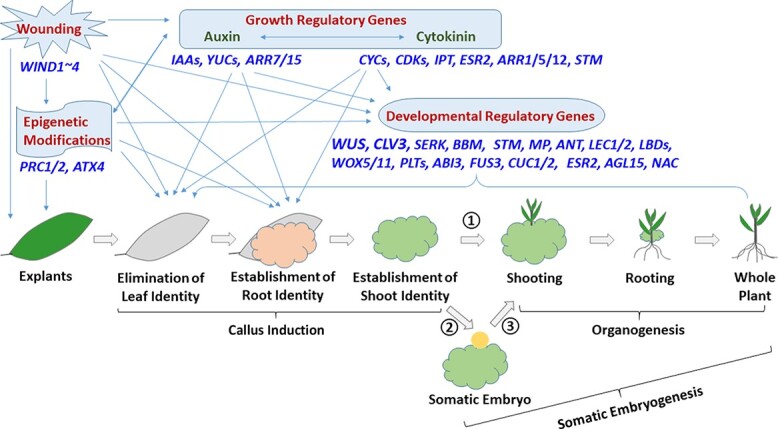
Key steps and factors in exogenous hormone-induced plant regeneration. Aerial explants go through the sequential steps of elimination of leaf identity, establishment of root identity, establishment of shoot identity, followed by organogenesis (step ①) or somatic embryogenesis (steps ② and ③). Wounding, epigenetic modifications, growth regulatory genes, and developmental regulatory genes are the four classes of crucial regeneration-promoting factors. Boxed arrows, key steps. Dark red, regeneration-promoting factors. Blue, key genes for each factor. Green, plant growth hormones. Orange circle, somatic embryo.

Through somatic embryogenesis and *de novo* organogenesis, transformed regeneration-competent cells develop into somatic embryos and shoot/root apical meristems (SAMs/RAMs), respectively, which develop into plantlets [6–12] (Figure 1). Somatic embryogenesis starts with the formation of an embryo-like structure from embryogenic cells like those in calli that further develops into a whole plant. Organogenesis may result in the formation of shoots and roots directly from explants (i.e. direct organogenesis) or start with the formation of SAM development from callus, and then the SAM develops into shoots that subsequently generate roots (i.e. indirect organogenesis) [13].

Since various factors affect callus formation and plant regeneration, conventional plant transformation requires optimizing many external factors including explant types, plant growth regulators (mainly auxin and cytokinin that are natural or synthetic chemicals exogenously applied to modify plant growth), basal media composition, pH, light conditions, and transgenic plant selection strategies. However, recent advances in the understanding and identification of plant growth and developmental regulatory genes (also called morphogenic genes) have revealed that many of these genes are involved in the regulation of biosynthesis and signaling pathways of auxin and cytokinin and thus control the plant regeneration process [2–5,10,12,14] (Figure 1; Table 1). Plant growth regulatory genes are involved in the biosynthesis, perception and transduction of plant growth hormones that are endogenously biosynthesized within plants and regulate plant growth. Plant developmental regulatory genes are the transcriptional factors or signaling molecules that control cell fate and thus regulate plant development (e.g. organ formation) by regulating the expression of various genes. The use of growth and developmental regulatory genes could significantly improve regeneration/transformation efficiency, speed up the transformation process, and enhance the application of gene editing in various crops [15–18] (Table 1). This review will discuss: *i*) recent advances in the identification of the functions of growth and developmental regulatory genes and their use in plant transformation; *ii*) the potential of these advances to create novel approaches for genotype-independent plant transformation and regeneration; and *iii*) how these advances can be harnessed to provide a better understanding of plant transformation from the molecular and developmental biology perspectives.

**Table 1:** Summary of plant growth and developmental regulatory genes studied in plant transgenic research

**Gene Cassette**	**Transformed Species**	**Explant**	**Hormone**	**Regeneration** ^**a**^	**Regenerated Plant**	**Transform. Efficiency**	**Abnormal Phenotype**	**Ref**
*Tissue culture – based plant transformation*								
*35S:AtWUS*	*Gossypium hirsutum*	Hypocotyl	2,4-D, kinetin	Embryogenesis	×	N.A.	√	[23]
*P_G10–90_:XVE-O^LexA^ -min35S:AtWUS*	*Nicotiana tabacum*	Leaf	BAP, NAA	Organogenesis	√	N.A.	√	[24,25]
	*Coffea canephora*	Leaf	BAP, IAA	Embryogenesis	√	N.A.	√	[26,27]
*P_G10–90_:XVE O^LexA^ -min35S:AtWOX5* or *2/8* or *2/9*	*N. tabacum*	Leaf	BAP, NAA	Organogenesis	√	N.A	√	[24,28]
*35S:GAL4 -AtRKD4-GR*	*Phalaenopsis*	Leaf	–	Embryogenesis	√	↑ 3.6 times	×	[29]
*35S:AtBBM; 35S:BnBBM*	*N. tabacum*	Leaf	IAA	Organogenesis	√	N.A.	√	[30,31]
*35S:BnBBM; HaUbi:BnBBM*	*Brassica napus*	Microspore	N.A.	Embryogenesis	×	N.A.	√	[32]
*35S:TcBBM*	*Theobroma cacao*	Cotyledon^b^	2,4-D, TDZ, kinetin	Embryogenesis	×	N.A.	√	[33,34]
*35S:TcBBM-GR*	*T. cacao*	Cotyledon^b^	2,4-D, TDZ, kinetin	Embryogenesis	×	N.A.	×	[35]
35S:*AtBBM-GR; 35S:BnBBM-GR*	*N. tabacum*	Leaf	IAA	Organogenesis	√	N.A.	×	[30, 31]
35S:*BnBBM-GR*	*Capsicum annuum*	Cotyledon	TDZ	Organogenesis	√	↑	√	[36]
*AtHSP18.2:FLP -35S:BcBBM*	*Populus tomentosa*	Leaf	NAA, zeatin	Embryogenesis	√	↑	×	[37]
*ZmUbi:ZmBBM* + *NOS:ZmWUS2*	*Zea mays*; *Oryza sativa*; *Sorghum bicolor*; *Saccharum officianrum*	Immature embryo	2,4-D, BAP	Embryogenesis	√	↑	×	[14]
*ZmUbi:ZmBBM* + *NOS:ZmWUS2*	*Z. mays*; *S. bicolor*	Immature embryo	2,4-D	Embryogenesis	√	↑	×	[16]
*ZmPLTP:ZmBBM* + *ZmAxig1:ZmWUS2*	*Z. mays*	Immature embryo	2,4-D, BAP	Embryogenesis	√	↑	×	[38]
*ZmPLTP:ZmWUS2*	*Z. mays*	Immature embryo	2,4-D, BAP	Embryogenesis	√	↑	×	[39]
*ZmPLTP:ZmBBM+ ZmPLTP:ZmWUS2*	*S. bicolor*	Immature embryo	2,4-D	Embryogenesis	√	↑	×	[40]
*35S:GVG-6xUAS -min35S:CcSERK1*	*C. canephora*	Leaf	NAA, BAP, kinetin	Embryogenesis	×	N.A.	×	[41]
*35S: PaHAP3A; P_G10–90_:XVE-O^LexA^ -min35S:PaHAP3A*	*Picea abies*	Embryonic cell lines	2,4-D, BA, NAA, ABA, IBA	Embryogenesis	×	N.A.	×	[42]
*35S:TcLEC2-GR*	*T. cacao*	Cotyledon^b^	2,4-D, TDZ, kinetin	Embryogenesis	×	N.A.	×	[35]
*P_G10–90_:XVE-O^LexA^ -min35S:AtLEC2*	*N. tabacum*	Leaf	BAP, NAA	Organogenesis	√	N.A.	√	[24,25]
*35S:GmAGL15*	*Glycine max*	Cotyledon	2,4-D	Embryogenesis	√	↑ 2 times	√	[43]
*35S:GhAGL15*	*G. hirsutum*	Hypocotyl	2,4-D, IAA, kinetin	Embryogenesis	×	×	×	[44]
*35S:BnSTM*; *35S:BoSTM*	*B. napus*	Hypocotyl	BAP, NAA	Embryogenesis	N.A.	N.A.	N.A.	[45]
*35S:NtNTHs*	*N. tabacum*	Leaf	BAP, NAA	Organogenesis	√	N.A.	√	[46]
*35S:ZmKn1*	*N. tabacum*	Leaf	–	Organogenesis	√	↑ 3 times	√	[47]
	*Citrus sinensis*	Internode	BAP, NAA, 2,4-D	Organogenesis	√	↑ 3 ~ 15 times	√	[48]
*35S:AtCUC1 or 2*	*Arabidopsis thaliana*	Seedling	IBA, IAA, 2,4-D, kinetin	Organogenesis	√	↑ 10 times	√	[49, 50]
*P_G10–90_:XVE-O^LexA^ -min35S:AtESR1*	*A. thaliana*	Root	2,4-D, IAA, 2-iP, kinetin	Organogenesis	√	↑	×	[51]
*P_G10–90_:XVE-O^LexA^ -min35S:AtESR2*	*A. thaliana*	Root	2,4-D, IAA, 2-iP, kinetin	Organogenesis	√	↑ 3 times	×	[52,53]
*AtMP:AtMP∆*	*A. thaliana*	Root, leaf, petiole, cotyledon	2,4-D, IBA, 2-iP	Organogenesis	√	N.A.	√	[54]
*2 × 35S:AtGRF5; 2 × 35S:BvGRF5-L*	*Beta vulgaris*	Cotyledon, hypocotyl	BAP, NAA	Organogenesis	√	↑ 6 times	×	[55]
*2 × 35S:AtGRF5; 2 × 35S:HaGRF5-L*	*Helianthus annuus*	Cotyledon	BAP, NAA	Organogenesis	√	↑	×	[55]
*PcUbi4–2*:: *GmGRF5-L*	*G. max*	Primary node	IAA, kinetin, IBA, zeatin	Organogenesis	√	↑	×	[55]
*PcUbi4–2*: *BnGRF5-L*	*B. napus*	Hypocotyl	2,4-D, zeatin, kinetin	Organogenesis	√	↑	×	[55]
*BdEF1:AtGRF5; BdEF1:ZmGRF5- L1/2*	*Z. mays*	Immature embryo	2,4-D, zeatin, IBA, BAP	Organogenesis	√	↑	×	[55]
*ZmUbi:GRF4-GIF1* *	*Triticum aestivum*	Immature embryo	2,4-D, zeatin	Organogenesis	√	↑ 7.8 times	×	[56]
	*O. sativa*	Seed	2,4-D, BAP, NAA	Organogenesis	√	↑ 2.1 times	×	
	*C. lemon*	Etiolated epicotyl	BAP, NAA, BA	Organogenesis	√	↑ 4.7 times	×	
*35S:ipt in Ac* *	*N. tabacum*	Leaf	–	Organogenesis	√	N.A.	×	[57]
	*P. sieboldii × P. grandi-entata*	Stem	IBA	Organogenesis	√	N.A.	×	
*35S:GVG - 6 × UAS -min35S:pt* *	*N. tabacum;*	Leaf	BAP, NAA	Organogenesis	√	↑ 1.2 times	×	[58]
	*Lactuca sativa*	Cotyledon	NAA	Organogenesis	√	↑ 3.8 times	×	
*Non-tissue culture – based plant transformation*								
*Nos:ZmWUS2 -ZmUbi:IPT* *; *Nos:ZmWUS2* -ZmUbi:AtSTM*	*A. thaliana; N. tabacum; Solanum* *lycopersicum*	Agro transient	–	Organogenesis	√	N.A.	×	[59]
	*S. tuberosum; N. tabacum; Vitis* *vinifera*	Mature plant	–	Organogenesis	√	N.A.	×	

a Embryogenesis, somatic embryogenesis.

b Staminodes were used as the explants to induce somatic embryos, and cotyledons from the somatic embryos were used for plant transformation.

c Antibiotic selectable marker-free. √, yes. ×, no.

### Key steps and factors in CIM-induced plant regeneration

Exogenous hormone-induced regeneration from aerial explants starts with the elimination of leaf identity in above-ground or aerial explants [19, 20] and then root and shoot identities [21, 22] needs to be re-established in both aerial and root explants, followed by *de novo* plant regeneration via somatic embryogenesis or organogenesis (Figure 1). The elimination of leaf identity is mainly achieved via epigenetic changes (see below). The establishment of lateral root identity occurs in pericycle-like cells of aerial explants or xylem-pole pericycle cells of root explants, permitting the middle cell layer of the pericycle-like cells to obtain pluripotency and develop into pericycle founder cells for callus induction [14, 22]. This includes the activation of expression of *ABERRANT LATERAL ROOT FORMATION4* (*ALF4*), the gene required for the first asymmetric division of pericycle cells during lateral root initiation, and RAM developmental regulatory genes such as *PLETHORA1* (*PLT1*) and *PLT2*, *WUSCHEL-RELATED HOMEOBOX5* (*WOX5*), *SHORT-ROOT* (*SHR*), and *SCARECROW* (*SCR*) [14]. Kareem et al. [21] found that transcription factors *PLT3*, *PLT5*, and *PLT7* activate the expression of *PLT1* and *PLT2* to establish pluripotency. Moreover, *PLT3*, *PLT5*, and *PLT7* activate the expression of the shoot-promoting factor *CUP-SHAPED COTYLEDON2* (*CUC2*) via *PLT*-mediated upregulation of auxin biosynthesis genes *YUCCA1* (*YUC1*) and YUC4, leading to shoot regeneration [60].

There are many genetic and environmental factors that can affect plant regeneration; among them, these four types of regeneration-promoting factors play essential roles: wounding, epigenetic modifications, growth regulatory genes, and developmental regulatory genes (Figure 1). By inducing a series of physical and chemical changes in the detached explants, wounding is the primary external trigger of callus induction from explants [5, 14]. These changes start with the perception of damage-associated molecular patterns such as extracellular ATP [61, 62] and cell wall-derived oligogalacturonic acid [63], triggering cytoplasmic calcium signaling and a burst of reactive oxygen species [61, 62]. These local wounding signals are translated into long-distance signals such as the electrical signal of cation channel GLUTAMATE RECEPTOR-LIKEs, inducing epigenetic modifications, alternations in the synthesis and accumulation of cytokinin and free auxin, and transcriptional upregulation of growth and developmental regulatory genes [10, 64] (Figure 1). Transcriptional changes include the activation of expression of callus-inductive chromatin remodeling regulator genes *POLYCOMB REPRESSIVE COMPLEX2* (*PRC2*; see below), cell cycle genes *CYCLINs* (*CYCs*) and *CYCLIN-DEPENDENT KINASES* (*CDKs*), cytokinin biosynthesis gene *ISOPENTENYL TRANSFERASE* (*IPT*), auxin biosynthesis gene *YUC5*, and *AP2*/*ERF* transcription factors *WOUND-INDUCED DEDIFFERENTIATION1 ~ 4* (*WIND1 ~ 4*) and *ENHANCER OF SHOOT REGENERATION1* (*ESR1*) [64, 65]. Among these, activation of expression of *CYCs* and *CDKs* triggers cells to reenter the cell cycle and reacquire cell proliferative competence, a central mechanism of callus induction [66], whereas WINDs promote callus induction by directly binding to the promoter of *ESR1* and upregulating its expression [67].

Epigenetic modifications of chromatin structure cause genome-wide changes in gene expression required for callus induction [11, 19, 20, 68]. This global reprogramming of epigenetic modifications includes changes in genome-wide DNA methylation (especially gene promoter DNA methylation), histone modifications in transcription start sites (TSSs) and other gene parts, and deposition of histone variants. For example, He et al. [19] discovered that genome-wide reprogramming of histone H3 lysine 27 trimethylation (H3K27me3) is critical in the leaf-to-callus transition since the *Arabidopsis thaliana prc2* (a key gene in establishing H3K27me3) mutants were defective in callus formation. The PRC2-mediated global epigenetic changes are directly involved in the elimination of the leaf identity in aerial explants by silencing leaf-regulatory genes while removing the repressive methyl marks on auxin pathway genes *YUC4* and *AUXIN/INDOLE3-ACETIC ACID2* (*AUX/IAA2*) and root-regulatory genes *WOX5* and *SHR* [19]. PRC2-dependent repressive histone modifications also control expression of wounding-responsive *WIND* genes [69], which promote callus induction by activating the cytokinin biosynthesis pathway via the upregulation of type-B *ARABIDOPSIS RESPONSE REGULATOR* (*ARR-B*) gene expression [70]. Lee et al. [20] found that AUXIN RESPONSE FACTOR7/9 (ARF7/9) and JUMONJI C DOMAIN-CONTAINING PROTEIN 30 (JMJ30) form the ARF-JMJ30 complexes to remove the methyl groups from H3K9me3 at *LATERAL ORGAN BOUNDARIES-DOMAIN16* (*LBD16*) and *LBD29*, leading to the activation of the expression of *LBD16* and *LBD29* for the establishment of lateral root identity. Lee et al. [71] revealed that the expression of chromatin modifier *ARABIDIPSIS TRITHORAX4* (*ATX4*) is repressed during callus induction to eliminate leaf identity but is reactivated to facilitate shoot identity establishment by removing the methyl groups from H3K4me3 at shoot identity genes such as *KNOTTED1-LIKE HOMEOBOX GENE 4* (*KNAT4*) and *YABBY 5* (*YAB5*). In addition, epigenetic reprogramming also provides local changes in the epigenetic states of key genes involved in callus induction and plant regeneration. These key genes include *WIND3*, *BABY BOOM* (*BBM*), *LEAFY COTYLEDON1* (*LEC1*) and *LEC2*, and *WOX5/11* genes [11]. All of these epigenetic modifications prepare explants for callus induction and are involved in all of the steps of callus induction and plant regeneration (Figure 1).

Cytokinins and auxins are critical for and routinely used in callus induction and plant regeneration. A balanced cytokinin and auxin ratio promotes callus induction, while high and low cytokinin-to-auxin ratios tend to induce shoot and root formation, respectively [72]. For example, endogenous auxin production and enhanced cytokinin sensitivity promote pluripotency acquisition in the middle cell layer of pericycle cells for organ regeneration [22]. In the middle cell layer of pericycle cells, endogenous auxin production increases through induced expression of *TRYPTOPHAN MONOTRANSFERASE OF ARABIDOPSIS1* (*TAA1*) by WOX5, PLT1 and PLT2, while cytokinin sensitivity increases through repression of *ARRs-A* by WOX5 and type-B ARR12. Hu et al. [73] also found that elevated endogenous auxin levels in the basal end of citrus epicotyl cuttings inhibit *in vitro* shoot organogenesis in a cytokinin-dependent manner. Similarly, genetic components of the biosynthesis and signaling pathways of cytokinin and auxin regulate callus formation and plant regeneration [54, 74–77]. For example, cytokinin induces expression of the D-type cyclin *CYCD3* gene, whose overexpression induces callus formation in the absence of cytokinin [78]. Cytokinin also induces expression of the transcription factor gene *SHOOT MERISTEMLESS* (*STM*), whose protein maintains cell division and inhibits cell differentiation in SAM and enhances cytokinin levels via activation of *IPT7* [79]. The overexpressed *A. tumefaciens IPT* gene in transgenic tobacco and cucumber induces cytokinin biosynthesis, resulting in the promotion of shoot organogenesis [77]. In addition, YUC-mediated auxin biosynthesis increases the total auxin level in leaf explants, and when polar auxin transport delivers auxin to regeneration-competent cells, it triggers *WOX11* and *WOX12* expression [74, 80]. WOX11 and WOX12 directly bind to the promoters of *WOX5* and *WOX7* and induce their expression for rapid root primordia initiation and root identity establishment [74, 80]. Auxin also inhibits the expression of type-A *ARR7* and *ARR15* via the auxin response transcription factor *MONOPTEROS* (*MP*) [ 81]. ARR-As negatively regulate *ARR-Bs* expression and thus repress cytokinin-mediated signaling [82].

**Figure 2:**
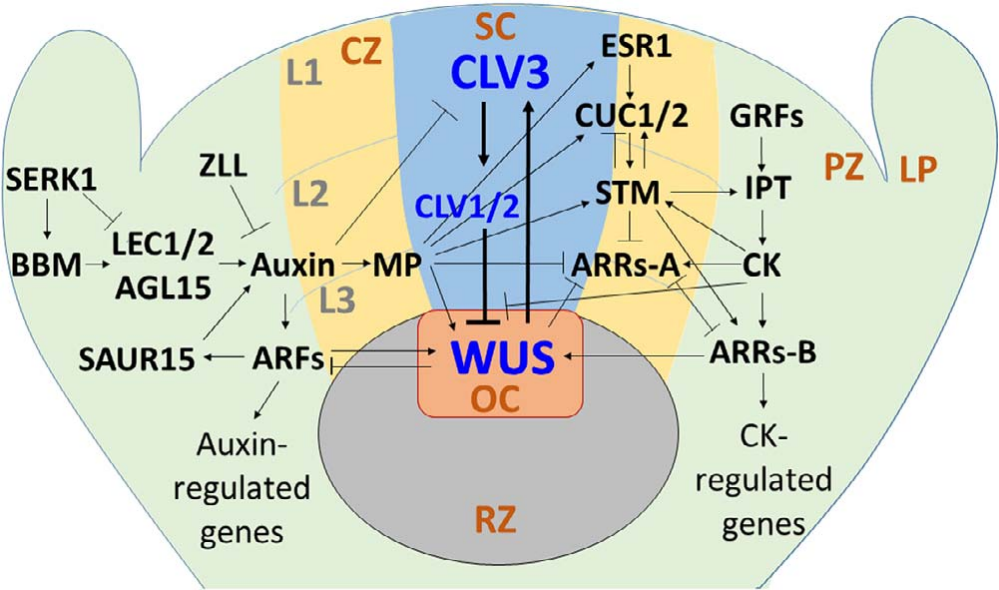
Shoot apical meristem (SAM), the WUS-CLV3 negative feedback loop, and the growth and developmental regulatory genes currently known to improve plant transformation efficiency. LP, leaf primordia; PZ, peripheral zone; CZ, central zone; SC, stem cells; OC, organizing center; RZ, rib zone; L1 – L3, cell layer 1–3.

Plant developmental regulatory genes also play key roles in callus formation and plant regeneration, as these processes are orchestrated by the sequential and spatiotemporal expression of various developmental regulatory genes driving morphogenesis [2–4, 83]. The most well-known developmental regulatory gene is *WUSCHEL* (*WUS*), the first gene identified in the *WOX* gene family and the essential player in both organogenesis [84] and embryogenesis [85]. As a homeodomain transcription factor, WUS is synthesized in the organizing center (OC) of the SAMs and migrates into the central zone (CZ) where it activates *CLAVATA3* (*CLV3*) transcription, which in turn inhibits *WUS* expression in the OC [86, 87] (Figure 2). The WUS-CLV3 negative feedback circuit regulates cell identity and maintains the existence of the OC and the shoot stem cell niche in the CZ (Figure 2). Su et al. [88] revealed that the activation of *WUS* transcription by auxin gradients results in the induction of embryogenic callus and somatic embryogenesis in *Arabidopsis* explants under *in vitro* culture conditions. Zhang et al. [89] demonstrated that the activation of *WUS* expression in *Arabidopsis* explants by the ARR-Bs/HD-ZIP III transcription factor complex in a cytokinin 2-isopentenyladenine (2-IP)-rich environment promotes organogenesis and shoot regeneration. As a result, both auxin and cytokinin activate *WUS* expression (Figure 1). In addition, various developmental transcription factor genes are involved in organogenesis and/or embryogenesis. These include *STM* [84,90], *MP*/*ARF5* [91,92], *LEC1/2* [93,94], *CUC1* [90], *ABSCISIC ACID-INSENSITIVE3* (*ABI3*) [94, 95], *FUSCA3* (*FUS3*) [94, 96], *AINTEGUMENTA* (*ANT*) [97], *WOX12* [80], and *CYCD3* [98].

**Figure 3:**
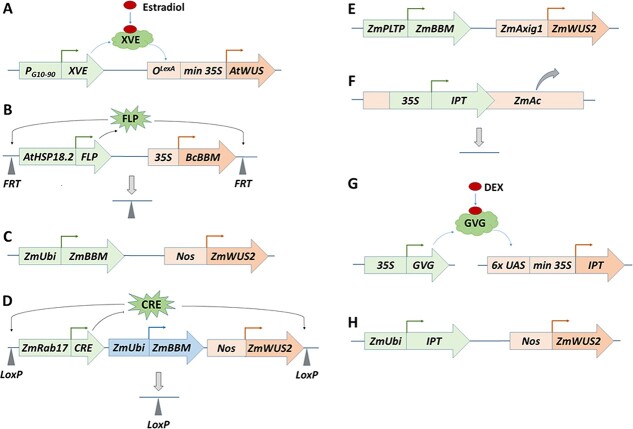
Structures and mechanisms of the expression vectors that utilized growth and developmental regulatory genes in plant transformation. (A) Estradiol-inducible *AtWUS* expression for transformation of *Coffea canephora* [26]. The *XVE* fusion gene driven by the constitutive *P_G10–90_* promoter contains the DNA-binding domain of the bacterial repressor LexA, the activation domain of the herpes viral protein VP16, and the carboxyl region of the human estrogen receptor. The binding of the estrogen hormone to the estrogen receptor in XVE enables XVE to bind to *O^LexA^*, the eight copies of the LexA operator sequence, leading to expression of *AtWUS* driven by *O^LexA^* and a minimal *35S* promoter. (B) A heat shock inducible-excision system to control *BcBBM* expression in transgenic Chinese white poplar [37]. Heat shock treatment on the stem cuttings of transgenic Chinese white poplar activates expression of the yeast *FLP* recombinase that is driven by the heat shock-inducible promoter *AtHSP18.2*, leading to the removal of the *AtHSP18.2:FLP* and *35S:BcBBM* cassette with one footprint (a single FRT recombination site) left in the transgenic genome. (C) Low expression of *ZmWUS2* under the control of the weak *Agrobacterium nopaline synthase* promoter (*Nos:ZmWUS2*) and high expression of *ZmBBM* driven by the strong maize *Ubiquitin* promoter (*ZmUbi:ZmBBM*) for transformation of maize and sorghum [15]. (D) A desiccation-inducible excision system to control *Nos:ZmWUS2* and *ZmUbi:ZmBBM* expression in transgenic maize and sorghum [15]. Desiccation of the embryogenic calli activates the expression of the *CRE* recombinase driven by the desiccation-inducible *ZmRab17* promoter, leading to the removal of *Nos:ZmWUS2*, *ZmUbi:ZmBBM* and *ZmRab17:CRE*, with one footprint (a single LoxP recombination site) left in the transgenic genome. (E) Conditional expression of the *ZmWUS2* and *ZmBBM* by the auxin-inducible promoter *ZmAxig1* and the maize embryo/leaf-specific promoter *ZmPLTP*, respectively, for maize transformation [38]. (F) A selectable marker-free transformation system in tobacco and hybrid aspen [57]. The *35S:IPT*-containing *Ac* transposase gene can automatically jump out of the chromosome, leaving no footprint in the transgenic genome. (G) Dexamethasone (Dex)-inducible *IPT* expression for transformation of tomato and lettuce [58]. The *GVG* fusion gene driven by the *35S* promoter contains the DNA-binding domain of the yeast transcription factor *GAL4*, the activation domain of VP16, and the hormone-binding domain of the rat *Glucocorticoid Receptor* (GR). Exogenous application of the synthetic glucocorticoid DEX releases GVG into the nucleus, where it binds to the *6× UPS* binding sites of GAL4 and activates the expression of *AtWUS* driven by *6× UPS* and a minimal *35S* promoter. (H) Low expression of *ZmWUS2* (*Nos:ZmWUS2*) plus high expression of the *Agrobacterium IPT* (*ZmUbi:IPT*) for organogenesis in the seedling leaves of *Arabidopsis*, tobacco, and tomato, and in the mature plants of tobacco, potato and grape [59].

These regeneration-promoting factors work together to control each step of callus formation and plant regeneration tightly and precisely (Figure 1). However, how these factors cooperate in plant regeneration is largely unknown.

### The use of plant developmental regulatory genes in plant transformation

As the most important developmental regulatory gene for the maintenance of the stem cell niche in SAMs, *WUS* has been used for plant transformation and regeneration in multiple species. Ectopic or estradiol-inducible expression of the *A. thaliana WUS* (*AtWUS*) gene (Figure 3A) in transgenic *Arabidopsis*, tobacco, *Gossypium hirsutum*, and *Coffea canephora* induced vegetative-to-embryogenic transition in vegetative tissues, which could differentiate into somatic embryos [23, 24, 26, 85] or organs [84]. However, the resulting transgenic plants exhibited abnormal phenotypes such as coiled root tips, cotton-like root structures, swollen hypocotyls, and distorted leaves [24, 26]. These abnormalities indicate *WUS* expression needs to be under a tight control since both cytokinin and auxin signaling pathways regulate its expression and its continuous overexpression causes malformations and alterations in transgenic plant growth. *WOX* genes such as *AtWOX2/5/8/9* have also been used individually for tobacco transformation in estradiol-inducible systems, resulting in transgenic plants with abnormal phenotypes such as dwarf plants or bulbous roots [28]. Since CLV1 works as the receptor for the secreted ligand CLV3 that negatively regulates *WUS* expression in the OC (Figure 2), RNAi-mediated silencing of the *Brassica napus CLV1* led to an increase in genetic transformation efficiency in transgenic *B. napus* and triggered a bushy phenotype [99]. Therefore, strategies are needed to fine-tune *WUS*/*WOXs* and *CLV1* expression to obtain normal transgenic plant regeneration (see below).

*BBM*, an *APETALA2 (AP2)/ETHYLENE RESPONSIVE ELEMENT BINDING FACTOR* (*AP2/ERF*) transcription factor, is another developmental regulatory gene involved in somatic embryogenesis and organogenesis via the auxin signaling pathway [32, 100, 101] (Figure 2). Khanday et al. [100, 102] reported that exogenous auxin-induced somatic embryogenesis in rice requires the presence of functional rice *BBM* (*OsBBM*) genes and the overexpression of *OsBBM1* promotes somatic embryogenesis without the use of exogenous auxins, suggesting the *OsBBM* overexpression increases endogenous auxin production. Moreover, overexpression of *BBM* genes activates somatic embryo formation and/or regeneration in transgenic tobacco [30], *B. napus* [32], and *Theobroma cacao* [33]. However, like the side effects of *WUS/WOXs* overexpression*,* plants overexpressing *BBMs* exhibit pleiotropic phenotypes such as mild-to-severe alterations in leaf and flower morphology.

A dexamethasone (Dex)-inducible expression system has also been used to drive the expression of *BBM* genes, which were fused in-frame with the hormone-binding domain of the rat glucocorticoid receptor (*GR*) gene [30, 35]. This resulted in transgenic tobacco [30] and *T. cacao* [35] with normal phenotypes, but caused thickened roots, pronounced apical hooks, and swelled cotyledons in transgenic *Capsicum annuum* [36]*.* Moreover, Deng et al. [37] used an inducible excision system (Figure 3B) to control the *B. campestris BBM* (*BcBBM*) overexpression in the calli of Chinese white poplar (*Populus tomentosa*). The site-directed recombination system containing the recombinase flippase and flippase recognition target (*FRT*) sites (FLP*/FRT*) from yeast (*Saccharomyces cerevisiae*) was under the control of the *Arabidopsis* heat shock-inducible promoter *AtHSP18.2*. Heat shock treatment on the transgenic stem cuttings caused the removal of the *AtHSP18.2:FLP* and *35S:BcBBM* cassette (Figure 3B) and produced transgenic plants with normal phenotypes. It was expected that high expression of *BBM* induces somatic embryogenesis while low expression of *BBM* promotes organogenesis as reduced cell differentiation was observed in low expression lines in transgenic Arabidopsis [94].

The upstream and downstream genes in the *BBM*-regulated somatic embryogenesis developmental pathway have also been examined for their roles in plant transformation. *SOMATIC EMBRYOGENESIS RECEPTOR KINASE1* (*SERK1*), a leucine-rich repeat receptor-like kinase (*LRR-RLK*) gene, regulates somatic embryogenesis by early activation of auxin biosynthesis, leading to the activation of expression of *WUS*, *BBM*, and the MADS-box transcription factor *AGAMOUS-LIKE15* (*AGL15*) as well as to the repression of expression of *LEC1* [41]. *CcSERK1* has been used in *C. canephora* transformation, but attempts to generate transgenic plants were unsuccessful [41]. Transcription factors LEC1 and LEC2, two downstream proteins in the *BBM*-regulated somatic embryogenesis development pathway [94], work redundantly with LEC1-LIKE (L1L) and the B3 domain proteins ABI3 and FUS3 in embryogenesis [103]. *LEC1* and *LEC2* have been used for transformation of *Picea abies* [42], *T. cacao* [35], and tobacco [24], and the estradiol-inducible expression of *AtLEC2* resulted in the regeneration of transgenic tobacco plants with curved root tips [24]. Since LEC2 directly activates the expression of *AGL15*, Thakare et al. [43] found that overexpression of *GmAGL15* increases soybean transformation efficiency by two-fold but resulted in abnormal phenotypes in the transgenic plants. Small auxin-upregulated RNA15 (SAUR15) is an auxin-inducible negative regulator in embryogenic callus induction, and its homozygous Mu transposon insertion mutant in maize (*zmsaur15*) exhibited 5 times higher transformation efficiency than the wild-type maize [104].

A recent groundbreaking study was published for monocot transformation through somatic embryogenesis by fine-tuning the expression of *WUS* and *BBM* [15]. Low expression of the maize *WUS2* gene by the *Agrobacterium* nopaline synthase promoter (*Nos:ZmWUS2*), a weak promoter for monocots, and high expression of the maize *BBM* by the strong maize *Ubiquitin* promoter (*ZmUbi:ZmBBM*) induced somatic embryogenesis and regeneration of fertile transgenic plants in immature embryos and/or callus of maize, sorghum, sugarcane and rice (Figure 3C). This approach significantly increased callus transformation efficiency, shortened regeneration time, and made various non-transformable genotypes transformable [15, 16]. For example, combined expression of *Nos:ZmWUS2* and *ZmUbi:ZmBBM* dramatically increased transformation efficiency from 0.0–2.0% to 25.3–51.7% in four transformation-recalcitrant maize inbred lines and made another 33 out of 50 commercially important Pioneer maize inbred lines transformable [15]. However, the continuous expression of both regulatory genes caused aberrant phenotypes such as stunted, twisted, sterile plants with thick, short roots. Lowe et al. [15] designed an inducible excision strategy to remove *Nos:ZmWUS2* and *ZmUbi:ZmBBM* in the transformed embryogenic calli by using the tyrosine recombinase CRE from the P1 bacteriophage and its recognition site *LoxP* (Figure 3D). Drying the embryogenic calli on filter paper for three days activated *CRE* expression driven by the desiccation-inducible maize promoter *ZmRab17*, leading to the removal of the transgenes (*WUS2*, *BBM*, and *CRE*) located between the two *LoxP* sites on the T-DNA (Figure 3D). The removal resulted in healthy, fertile T_0_ transgenic plants [15]. The effectiveness of this strategy was also confirmed in previously non-transformable maize and sorghum varieties [16, 17].

Moreover, Lowe et al. [38] replaced the *ZmUbi* promoter with a maize phospholipid transferase promoter *ZmPLTP* to drive *BBM* expression (Figure 3E); *ZmPLTP* has strong expression in maize embryos and leaves but low expression in ears and tassels, and undetectable expression in roots. Combined expression of *Nos:ZmWUS2* and *ZmPLTP:ZmBBM* in immature zygotic embryos induced the formation of somatic embryos in one week, which directly developed into healthy fertile plants without callus formation. This strategy significantly shortened the transformation process since the desiccation-inducible excision method in Lowe et al. [15] requires three months of callus induction. Since the T1 seeds continuously expressing *Nos:ZmWUS2* showed inconsistent germination, Lowe et al. [38] also replaced the *Nos* promoter with the maize auxin-inducible *ZmAxig1* promoter to drive *ZmWUS2* expression (Figure 3E). The expression levels of *ZmAxig1:ZmWUS2* and *ZmPLTP:ZmBBM* were specific and low in non-embryogenic and un-induced tissues under the control of these two maize promoters, resulting in rapid somatic embryo development without callus formation so that excision is not needed to generate phenotypically normal transgenic plants. The resulting callus-free transformation approach has made all seven tested maize genotypes highly transformable [38], indicating the primary function of WUS and BBM in cell proliferation other than callus formation. Interestingly, it was recently shown that *ZmPLTP:ZmWUS2* alone is sufficient to promote somatic embryogenesis and transformation of recalcitrant maize varieties in a non-cell autonomous fashion, i.e. *WUS2* expression in a transformed cell could stimulate somatic embryogenesis and plant regeneration in neighboring cells [39]. Hoerster et al. [39] conducted maize transformation by using the mixture of two strains of *A. tumefaciens* containing *ZmPLTP:ZmWUS2* or selectable and visual marker cassettes, and obtained T0 transgenic maize plants expressing the selectable marker gene but did not contain *ZmWUS2*. Similarly, Aregawi et al. [40] conducted sorghum transformation by using the mixture of two strains of *A. tumefaciens* with one containing *ZmPLTP:ZmWUS2* and *ZmPLTP:ZmBBM* and the other one containing selectable marker cassettes, and obtained T0 transgenic sorghum plants expressing the selectable marker gene but did not contain *ZmWUS2* and *ZmBBM*. This approach shortened sorghum transformation time by nearly half and made several previously untransformable genotypes transformable.

*STM*, a KNOX homeodomain transcription factor, is expressed in SAMs and prevents the differentiation of the meristematic cells [105]. STM induces expression of *IPT7*, a cytokinin biosynthesis gene, leading to an increased cytokinin level [79, 106] (Figure 1). Overexpression of the maize *STM* homolog *Knotted1* (*ZmKn1*) in transgenic citrus significantly increased citrus transformation efficiency 3 ~ 15 times [48]. *ZmKn1* overexpression in transgenic tobacco significantly increased transformation efficiency by 3 times via organogenesis on a hormone-free medium without antibiotic selection [47]. However, overexpression of *STM* homologs resulted in the regeneration of transgenic tobacco plants with a bushy phenotype [46, 47]. In addition, overexpression of *CUC1* and *CUC2* that positively regulate SAM formation via STM-dependent (and STM-independent) pathways resulted in enhanced transformation efficiency of *Arabidopsis* by 10 times via a tissue culture method, and the resulting transgenic plants showed phenotypic abnormalities [49]. The estradiol-inducible expression of *ESR2*, an *AP2*-domain transcription factor, also enhanced *Arabidopsis* tissue culture transformation by directly regulating *CUC1* transcription [52].

Transcription factor MP, a mediator of auxin responses and regulator of cytokinin signaling and biosynthesis, can also be leveraged to increase transformation efficiency [107, 108]. When the regulatory domain of *MP* is removed, the resultant *MPΔ* becomes irrepressible but maintains the normal *MP* function. Overexpression of *AtMPΔ* increased transformation efficiency in transgenic *Arabidopsis* with abnormal phenotypes via the upregulation of expression of *AtWUS*, *AtSTM*, *AtESR1*, *AtCUC1* and *AtCUC2* [54,107] and repression of type-A *ARR*5 and *ARR7* [81].

In contrast to the negative pleiotropic effects of overexpression of the aforementioned genes (i.e. *WUS*, *WOXs, BBM*, *SERK1*, *LEC1/2*, *AGL15, STM*/*Kn1*, *CUC1/2*, *ESR2,* and *MPΔ*) and silencing/knockout of *CLV1* and *SAUR15*, constitutive expression of a small family of transcription factor genes *GROWTH-REGULATING FACTOR* (*GRF*) and its transcriptional cofactor *GRF-INTERACTING FACTOR1* (*GIF*) does not cause observed undesirable phenotypes in transgenic plants [55, 56], making conditional expression or excision of the transgenes unnecessary. During callus induction and plant regeneration, the miR396-regulated GRF-GIF duo can recruit SWITCH/SUCROSE NONFERMENTING (SWI/SNF) chromatin remodeling complexes to regulate expression of their target genes and specify meristematic identity for organogenesis [109–111]. For example, poplar PpnGRF5–1 forms a complex with PpnGIFs and then inhibits expression of cytokinin oxidase/dehydrogenase1 (*PpnCKX1*), which is a membrane-bound protein catalyzing the degradation of cytokinins [112], leading to the accumulation of cytokinins and meristematic induction [113] (Figure 2). Kong et al. [55] found that the overexpression of *AtGRF5* or its homologs from various plant species enhanced shoot organogenesis and transformation efficiency in transformation-recalcitrant sugar beet, canola, soybean, and sunflower, and promoted somatic embryogenesis and transformation efficiency in maize. When compared to the control plants, a 4.5 ~ 11.5-, 1.9 ~ 2.3- and 1.1 ~ 1.4-fold increase in transformation efficiency was achieved in sugar beet, canola, and soybean, respectively. The resultant transgenic plants showed normal phenotypes. Debernardi et al. [56] demonstrated that overexpression of the wheat *GRF4-GIF1* chimeric gene dramatically increases transformation efficiency and regenerates fertile transgenic plants with normal phenotypes in multiple plant species without the use of exogenous cytokinins. These species included wheat, rice, citrus as well as some difficult- or recalcitrant-to-transform species/genotypes such as commercial durum, bread wheat and a triticale line [56]. Most importantly, it was shown that overexpression of *GRF4-GIF1* chimera increased the regeneration efficiency by an average of 7.8-fold and shortened the transformation process time from 91 days to 56 days in the wheat genotypes, permitting transgenic shoot selection in auxin media without using antibiotic selectable marker genes. Since there are multiple members in the *GRF-GIF* families, and not all GRFs or GRF-GIF pairs work equally effectively in plant transformation, research needs to be conducted to identify the GRFs or GRF-GIF pairs that have high transformation efficiency in a given economically important crop.

### The use of plant growth regulatory genes in plant transformation

The *IPT* gene from the Ti-plasmids of *A. tumefaciens* catalyzes the formation of isopentenyl-adenosine-5-monophosphate (isopentenyl-AMP), the first intermediate in the cytokinin biosynthesis pathway, resulting in elevated cytokinin levels [114, 115] (Figure 2). Overexpression of an *Agrobacterium IPT* gene enhanced cytokinin level 23 ~ 300 times, resulting in a 24 ~ 2,000-fold increase in the cytokinin-to-auxin ratios in tobacco and cucumber [116, 117]. The abnormal phenotypes of the transgenic plants included loss of apical dominance and a poor ability to root, reduced internode elongation, and altered leaf morphology [116–118]. To overcome the negative phenotypic effects, Ebinuma et al. [57] developed a selectable marker-free transformation system by inserting the *35S:IPT* cassette into an *Ac*-element from maize (Figure 3F) for transformation of tobacco and hybrid aspen (*Populus sieboldii × P.grandidentata*). Following the somatic self-excision of the *IPT*-containing *Ac*-element in the hemizygous transgenic lines where the retroelement failed to integrate into the sister chromatin, normal marker-free shoots were obtained at a frequency of 0.5 ~ 1.0%. Kunkel et al. [58] used the *IPT* gene for antibiotic marker-free tobacco and lettuce transformation by using a Dex-inducible *IPT* expression system (Figure 3G). The induced *IPT* expression improved transformation efficiency by 24.3 and 6.6 times in tobacco and lettuce, respectively, and produced transgenic plants without observed morphological defects.

Using *Agrobacterium*-mediated transient expression, a recent breakthrough in callus-free plant regeneration of multiple species was published [59]. This work revealed that low expression of *ZmWUS2* (*Nos:ZmWUS2*) plus high expression of the *Agrobacterium IPT* (*ZmUbi:IPT*) or *ZmUbi:AtSTM* (Figure 3H) promoted organogenesis in aseptically grown seedling leaves of *Arabidopsis*, tobacco, and tomato, and in mature plants of tobacco, potato and grape [59]. When used together with a Cas9/gRNA plasmid, this novel approach produced gene-edited shoots without the use of tissue culture, offering great potential to speed up the breeding cycles for many plant species [59]. This tissue culture-free approach provides a good tool to test the effects of different candidate genes and regulatory elements on plant transformation.

## Conclusion and future perspectives

With the demonstration of the dramatic effects of growth and developmental regulatory genes in plant tissue culture and transformation, regulatory genes have emerged as innovative, game-changing tools in plant transformation. These regulatory genes have been demonstrated to work efficiently in dicots, monocots and gymnosperms (Table 1) and are expected to work in various plants including specialty crops such as potato, sweetpotato, tomato and ornamentals. They offer excellent opportunities to develop genotype-independent genetic transformation methods and gene editing approaches in specialty crops.

In addition to the genes discussed above, many other upstream and downstream interacting factors promote meristem formation, shoot regeneration, or somatic embryogenesis, but have yet to be developed for use in plant transformation. These include *PLTs* [21], *WINDs* [65,119], *ARRs* [120], *ABI3* [121], *FUS3* [121], *LILs* [122], *AGL18* [123], *Pollen ole e 1* (*POE1*) [124], *EMBRYO SAC DEVELOPMENT ARREST* (*EDA40*) [124], *SUPERMAN* (*SUP*) [124, 125], and *AT-HOOK MOTIF CONTAINING NUCLEAR LOCALIZED 15* (*AHL15*) [126]. Research needs to be conducted to fine-tune the expression of each of these genes to examine their effects on plant regeneration and transformation.

Future research to identify additional growth and developmental regulatory genes and novel regulatory network looks promising. Various combinations of different growth and developmental regulatory genes need to be tested rationally for their synergistic and additive effects on plant transformation. Fine-tuning the expression of these genes is critical for the regeneration of normal, fertile plants in different plant species since their constitutive/ectopic expression typically interferes with normal plant growth and development and causes undesirable pleiotropic effects. Strategies to counter these pleiotropic effects include tissue- or growth stage-specific expression, inducible or conditional expression, or removal of the transgenes. Synthetic promoters or devices could be used to conditionally express the regulatory genes [127, 128]. T-DNA read through-based transient expression [129] is also worth further testing. In addition, protein or DNA-free delivery of the regulatory proteins could be explored for their effects on plant transformation and regeneration, which could be conducted in explants cultured on callus induction media, in protoplasts [130], or suspension cells [131]. Transient or protein delivery of these regulatory genes could enable DNA-free approaches for the generation of engineered crop cultivars, which will minimize regulations and public opposition. No matter the genes or the delivery and regeneration systems, the success of next-generation agriculture will greatly depend on our ability to expand transgenic capabilities to previously recalcitrant species and elite genotypes. The recent progress in this exciting area of plant science provides an optimistic outlook for the future of crop transformation.
